# Supplementary material for: Pyruvate Dehydrogenase Kinase 1 inhibition mediated oxidative phosphorylation enhancement in cartilage promotes osteoarthritis progression
Source: BMC Musculoskelet Disord. 2023 Jul 20;24:597. doi: 10.1186/s12891-023-06585-6 (PMC10357736; doi:10.1186/s12891-023-06585-6)
Supplement: Supplementary file 1 — Additional file 1. [file 12891_2023_6585_MOESM1_ESM.zip › KEGG.Gsea.1653622432464/KEGG_GLYCEROLIPID_METABOLISM.html]

Details for gene set KEGG\_GLYCEROLIPID\_METABOLISM[GSEA]

|  || Dataset | 55457\_collapsed\_to\_symbols.cls.cls #OA\_versus\_normal.cls.cls #OA\_versus\_normal\_repos |
| Phenotype | cls.cls#OA\_versus\_normal\_repos |
| Upregulated in class | OA |
| GeneSet | KEGG\_GLYCEROLIPID\_METABOLISM |
| Enrichment Score (ES) | 0.17459294 |
| Normalized Enrichment Score (NES) | 0.67042947 |
| Nominal p-value | 0.9516129 |
| FDR q-value | 1.0 |
| FWER p-Value | 1.0 |
Table: GSEA Results Summary

  

Fig 1: Enrichment plot: KEGG\_GLYCEROLIPID\_METABOLISM      
 Profile of the Running ES Score & Positions of GeneSet Members on the Rank Ordered List

  

| SYMBOL | TITLE | RANK IN GENE LIST | RANK METRIC SCORE | RUNNING ES | CORE ENRICHMENT || 1 | LIPC | "lipase C, hepatic type [Source:HGNC Symbol;Acc:HGNC:6619]" | 457 | 0.678 | 0.0195 | Yes |
| 2 | AKR1A1 | aldo-keto reductase family 1 member A1 [Source:HGNC Symbol;Acc:HGNC:380] | 808 | 0.567 | 0.0383 | Yes |
| 3 | AGPAT1 | 1-acylglycerol-3-phosphate O-acyltransferase 1 [Source:HGNC Symbol;Acc:HGNC:324] | 907 | 0.540 | 0.0746 | Yes |
| 4 | ALDH2 | aldehyde dehydrogenase 2 family member [Source:HGNC Symbol;Acc:HGNC:404] | 1003 | 0.517 | 0.1091 | Yes |
| 5 | PLPP1 | phospholipid phosphatase 1 [Source:HGNC Symbol;Acc:HGNC:9228] | 1109 | 0.494 | 0.1410 | Yes |
| 6 | ALDH3A2 | aldehyde dehydrogenase 3 family member A2 [Source:HGNC Symbol;Acc:HGNC:403] | 1263 | 0.465 | 0.1669 | Yes |
| 7 | ALDH7A1 | aldehyde dehydrogenase 7 family member A1 [Source:HGNC Symbol;Acc:HGNC:877] | 2158 | 0.343 | 0.1252 | Yes |
| 8 | DGKQ | diacylglycerol kinase theta [Source:HGNC Symbol;Acc:HGNC:2856] | 2516 | 0.302 | 0.1220 | Yes |
| 9 | DGKI | diacylglycerol kinase iota [Source:HGNC Symbol;Acc:HGNC:2855] | 2774 | 0.273 | 0.1241 | Yes |
| 10 | ALDH9A1 | aldehyde dehydrogenase 9 family member A1 [Source:HGNC Symbol;Acc:HGNC:412] | 2795 | 0.270 | 0.1445 | Yes |
| 11 | GK2 | glycerol kinase 2 [Source:HGNC Symbol;Acc:HGNC:4291] | 3042 | 0.246 | 0.1453 | Yes |
| 12 | AKR1B1 | aldo-keto reductase family 1 member B [Source:HGNC Symbol;Acc:HGNC:381] | 3138 | 0.236 | 0.1571 | Yes |
| 13 | PNLIPRP2 | pancreatic lipase related protein 2 (gene/pseudogene) [Source:HGNC Symbol;Acc:HGNC:9157] | 3268 | 0.224 | 0.1653 | Yes |
| 14 | AGK | acylglycerol kinase [Source:HGNC Symbol;Acc:HGNC:21869] | 3373 | 0.214 | 0.1746 | Yes |
| 15 | DGAT1 | diacylglycerol O-acyltransferase 1 [Source:HGNC Symbol;Acc:HGNC:2843] | 3950 | 0.160 | 0.1428 | No |
| 16 | MGLL | monoglyceride lipase [Source:HGNC Symbol;Acc:HGNC:17038] | 4018 | 0.154 | 0.1502 | No |
| 17 | GLA | galactosidase alpha [Source:HGNC Symbol;Acc:HGNC:4296] | 4172 | 0.140 | 0.1497 | No |
| 18 | TKFC | triokinase and FMN cyclase [Source:HGNC Symbol;Acc:HGNC:24552] | 4989 | 0.074 | 0.0922 | No |
| 19 | AGPAT2 | 1-acylglycerol-3-phosphate O-acyltransferase 2 [Source:HGNC Symbol;Acc:HGNC:325] | 5545 | 0.029 | 0.0514 | No |
| 20 | ALDH1B1 | aldehyde dehydrogenase 1 family member B1 [Source:HGNC Symbol;Acc:HGNC:407] | 6551 | -0.046 | -0.0231 | No |
| 21 | DGKB | diacylglycerol kinase beta [Source:HGNC Symbol;Acc:HGNC:2850] | 6801 | -0.063 | -0.0373 | No |
| 22 | DGKG | diacylglycerol kinase gamma [Source:HGNC Symbol;Acc:HGNC:2853] | 6938 | -0.075 | -0.0418 | No |
| 23 | DGKA | diacylglycerol kinase alpha [Source:HGNC Symbol;Acc:HGNC:2849] | 7570 | -0.125 | -0.0807 | No |
| 24 | DGKE | diacylglycerol kinase epsilon [Source:HGNC Symbol;Acc:HGNC:2852] | 8208 | -0.172 | -0.1163 | No |
| 25 | LPL | lipoprotein lipase [Source:HGNC Symbol;Acc:HGNC:6677] | 8374 | -0.184 | -0.1142 | No |
| 26 | CEL | carboxyl ester lipase [Source:HGNC Symbol;Acc:HGNC:1848] | 8375 | -0.184 | -0.0993 | No |
| 27 | PNPLA3 | patatin like phospholipase domain containing 3 [Source:HGNC Symbol;Acc:HGNC:18590] | 8455 | -0.190 | -0.0900 | No |
| 28 | AGPAT4 | 1-acylglycerol-3-phosphate O-acyltransferase 4 [Source:HGNC Symbol;Acc:HGNC:20885] | 8577 | -0.200 | -0.0832 | No |
| 29 | PNLIPRP1 | pancreatic lipase related protein 1 [Source:HGNC Symbol;Acc:HGNC:9156] | 8798 | -0.216 | -0.0828 | No |
| 30 | LIPF | "lipase F, gastric type [Source:HGNC Symbol;Acc:HGNC:6622]" | 9074 | -0.238 | -0.0848 | No |
| 31 | PLPP3 | phospholipid phosphatase 3 [Source:HGNC Symbol;Acc:HGNC:9229] | 9906 | -0.312 | -0.1241 | No |
| 32 | MBOAT2 | membrane bound O-acyltransferase domain containing 2 [Source:HGNC Symbol;Acc:HGNC:25193] | 10038 | -0.325 | -0.1079 | No |
| 33 | LIPG | "lipase G, endothelial type [Source:HGNC Symbol;Acc:HGNC:6623]" | 10181 | -0.339 | -0.0914 | No |
| 34 | GK | glycerol kinase [Source:HGNC Symbol;Acc:HGNC:4289] | 10291 | -0.350 | -0.0715 | No |
| 35 | DGKZ | diacylglycerol kinase zeta [Source:HGNC Symbol;Acc:HGNC:2857] | 10530 | -0.376 | -0.0595 | No |
| 36 | PLPP2 | phospholipid phosphatase 2 [Source:HGNC Symbol;Acc:HGNC:9230] | 11160 | -0.452 | -0.0717 | No |
| 37 | PNLIP | pancreatic lipase [Source:HGNC Symbol;Acc:HGNC:9155] | 11329 | -0.475 | -0.0462 | No |
| 38 | AGPAT3 | 1-acylglycerol-3-phosphate O-acyltransferase 3 [Source:HGNC Symbol;Acc:HGNC:326] | 12062 | -0.611 | -0.0535 | No |
| 39 | DGKD | diacylglycerol kinase delta [Source:HGNC Symbol;Acc:HGNC:2851] | 12222 | -0.651 | -0.0130 | No |
| 40 | DGKH | diacylglycerol kinase eta [Source:HGNC Symbol;Acc:HGNC:2854] | 12619 | -0.809 | 0.0218 | No |
Table: GSEA details [plain text format]

  

Fig 2: KEGG\_GLYCEROLIPID\_METABOLISM      
 Blue-Pink O' Gram in the Space of the Analyzed GeneSet

  

Fig 3: KEGG\_GLYCEROLIPID\_METABOLISM: Random ES distribution      
 Gene set null distribution of ES for **KEGG\_GLYCEROLIPID\_METABOLISM**
